# Characterization and influence of cardiac background sodium current in the atrioventricular node

**DOI:** 10.1016/j.yjmcc.2016.04.014

**Published:** 2016-08

**Authors:** Hongwei Cheng, Jue Li, Andrew F. James, Shin Inada, Stéphanie C.M. Choisy, Clive H. Orchard, Henggui Zhang, Mark R. Boyett, Jules C. Hancox

**Affiliations:** a School of Physiology, Pharmacology & Neuroscience, Biomedical Sciences Building, University of Bristol, Bristol BS8 1TD, UK; b Institute of Cardiovascular Sciences, University of Manchester, Core Technology Facility, 46 Grafton Street, Manchester M13 9NT, UK; c Department of Physics and Astronomy, University of Manchester, Oxford Road, Manchester M13 9PL, UK

**Keywords:** Atrioventricular node, AVN, Background current, I_B,Na_, Pacemaking

## Abstract

Background inward sodium current (I_B,Na_) that influences cardiac pacemaking has been comparatively under-investigated. The aim of this study was to determine for the first time the properties and role of I_B,Na_ in cells from the heart's secondary pacemaker, the atrioventricular node (AVN). Myocytes were isolated from the AVN of adult male rabbits and mice using mechanical and enzymatic dispersion. Background current was measured using whole-cell patch clamp and monovalent ion substitution with major voltage- and time-dependent conductances inhibited. In the absence of a selective pharmacological inhibitor of I_B,Na_, computer modelling was used to assess the physiological contribution of I_B,Na_. Net background current during voltage ramps was linear, reversing close to 0 mV. Switching between Tris- and Na^+^-containing extracellular solution in rabbit and mouse AVN cells revealed an inward I_B,Na_, with an increase in slope conductance in rabbit cells at − 50 mV from 0.54 ± 0.03 to 0.91 ± 0.05 nS (mean ± SEM; n = 61 cells). I_B,Na_ magnitude varied in proportion to [Na^+^]_o_. Other monovalent cations could substitute for Na^+^ (Rb^+^ > K^+^ > Cs^+^ > Na^+^ > Li^+^). The single-channel conductance with Na^+^ as charge carrier estimated from noise-analysis was 3.2 ± 1.2 pS (n = 6). Ni^2 +^ (10 mM), Gd^3 +^ (100 μM), ruthenium red (100 μM), or amiloride (1 mM) produced modest reductions in I_B,Na_. Flufenamic acid was without significant effect, whilst La^3 +^ (100 μM) or extracellular acidosis (pH 6.3) inhibited the current by > 60%. Under the conditions of our AVN cell simulations, removal of I_B,Na_ arrested spontaneous activity and, in a simulated 1D-strand, reduced conduction velocity by ~ 20%. I_B,Na_ is carried by distinct low conductance monovalent non-selective cation channels and can influence AVN spontaneous activity and conduction.

## Highlights

• A background sodium current, I_B,Na_, has been identified in cells from the atrioventricular node (AVN).
• AVN I_B,Na_ is carried by non-selective cation channels.
• The single channel conductance derived from ‘noise analysis’ is 3.2 ± 1.2 pS.
• In AVN cell simulations, removal of I_B,Na_ arrested spontaneous activity and reduced conductance in a 1D strand.

## 1 Introduction

The atrioventricular node (AVN) is normally the only site through which electrical activity can pass from atria to ventricles; slow conduction through the AVN facilitates completion of atrial contraction prior to that of the ventricles [1], [2], [3]. The filtering properties of the AVN can also serve a protective function during some supraventricular tachyarrhythmias [2], [3]. The AVN possesses pacemaking properties and should the sinoatrial node (SAN) fail or normal conduction become impaired, the AVN can take over pacemaking of the ventricles [2], [3]. In the heart's primary pacemaker, the sinoatrial node, the cellular basis of pacemaking is established to involve both calcium and membrane ‘clocks’, with spontaneous rate influenced by cellular Ca^2 +^ dynamics and by multiple sarcolemmal ionic currents [4], [5]. In contrast, the cellular electrophysiological basis of AVN pacemaking is incompletely understood, though it is clear that this is also likely to involve multiple ionic conductances [6], [7], [8]. For example, in the rabbit intact AVN inhibition of the hyperpolarization activated pacemaker current “I_f_” slows but does not stop AVN junctional rhythm [9], [10]; this is consistent with an important though not obligatory role for I_f_ in AVN pacemaking. There is also evidence from both rabbit and dog preparations that intracellular Ca^2 +^ cycling influences AVN pacemaking rate [11], [12], [13], [14], whilst Cav1.3 and 3.1 have been implicated in mouse AVN pacemaking [8].

The potential importance of a background inward conductance in SAN pacemaking has long been recognised and such a conductance was incorporated even in early models of SAN pacemaking (e.g. [15], [16], [17]). In 1990, Denyer and Brown provided strong, though indirect, evidence for a role for a background inward current in rabbit isolated SAN cell pacemaking during I_f_ inhibition with Cs^+^ [18]. A subsequent study by Hagiwara and colleagues provided direct evidence for a Na^+^-dependent background inward current (I_B,Na_) in SAN cells from the same species [19]. Much less is known in this regard for the AVN. Strong, albeit indirect, evidence that AVN cells possess a marked ‘resting’ permeability to one or more types of inwardly moving cation comes from the fact that voltage-clamped small AVN tissue preparations and AVN cells exhibit a ‘zero current’ potential of ~− 40 mV (e.g. [6], [20], [21], [22]), which is some distance from the K^+^ equilibrium potential. Spontaneously active AVN cells arrested by Ca^2 +^ channel blockade also exhibit a ‘resting’ potential of near ~− 40 mV [23]. However, no experimental data are available that address directly the nature of background inward current in the AVN. Moreover, uncertainties remain regarding the underlying basis for cardiac background inward current, with suggestions including that it might result from ‘leak’ forms of activity of the Na-K pump or Na-Ca exchange [24], [25]. Consequently, the present study was undertaken to determine whether or not AVN cells possess a cation current analogous to SAN I_B,Na_ and, if present, to determine its characteristics. The results of this study demonstrate that an I_B,Na_ is present in AVN cells and that it has the ability to make a substantial contribution to AVN cell electrophysiology. We also provide what, to our knowledge, is the first numerical estimate of single channel conductance for the channels underlying I_B,Na_ for any cardiac cell type, demonstrating that cardiac I_B,Na_ is carried by a distinct background cation channel.

## 2 Methods

### 2.1 AVN cell isolation

Male New Zealand White rabbits (2–3 kg) were killed humanely in accordance with UK Home Office legislation. AVN cells were isolated by enzymatic and mechanical dispersion as described previously [21], [26]. The AVN region within the Triangle of Koch was identified in relation to anatomical landmarks and removed for cell dispersion [21], [26]. AVN cells from male C57BL/6 mice (19–31 g) were isolated using a similar method, which is described in detail in [27]. Murine AVN cells were used to determine the presence of I_B,Na_ in AVN cells from an additional species to rabbit (Fig. 2). All experiments shown in other figures were performed on rabbit AVN cells. Cells were stored in refrigerated (4 °C) Kraftbrühe ‘KB’ solution [28] until use.

### 2.2 Electrophysiological recording

Cells were placed in an experimental chamber mounted on the stage of an inverted microscope (Eclipse TE2000-U, Nikon, Japan) and superfused with a Tyrode's solution containing (in mM) NaCl 136.9, KCl 5.4, NaH_2_PO_4_ 0.33, CaCl_2_ 1.8, MgCl_2_ 0.5, HEPES 5 and Glucose 5 (pH 7.4 with NaOH). Whole-cell patch-clamp recordings were made using an Axopatch-1D amplifier (Axon Instruments, USA). Patch-pipettes (A-M Systems, USA) were pulled and heat-polished to a final resistance of 2–3 MΩ (Narishige PP-83 and Narishige MF-83, Japan). Protocols were generated and data recorded on-line with pClamp 10.0 software (Axon instruments, USA) via an analogue-to-digital converter Digidata 1322 (Axon Instruments/Molecular Devices, USA). Membrane currents were recorded in whole cell voltage-clamp mode, with a digitization frequency of 10 kHz. For cation current recordings, the same solutions as employed in [29] were used: Na^+^-containing external solution contained (in mM) 150 NaCl, 5 HEPES, 2 CsCl, 2 NiCl_2_, 1 BaCl_2_, 1 MgCl_2_, 0.01 Strophanthidin (pH 7.4 with Tris base), whilst for Na^+^-free (Tris-substituted) solution, NaCl was replaced with equimolar Tris base (pH 7.4 with HCl). For experiments with various extracellular Na^+^ concentrations, NaCl and Tris base made a total concentration of 150 mM for each individual solution; but for experiments with a high [Na^+^]_o_ exceeding 150 mM (cf [29]) a solution containing 200 mM NaCl was used. For experiments involving monovalent substitution, NaCl was replaced with equimolar CsCl, LiCl, KCl or RbCl. For reduced pH extracellular solution, pH was set to 6.3 (with HCl). The pipette solution for background current recording contained (in mM): 120 CsOH, 20 CsCl, 5 HEPES, 10 EGTA, 5 K_2_-creatine phosphate, 5 Mg-ATP, 2 MgCl_2_, 100 aspartic acid (pH of 7.4 with CsOH) [29]. Once the whole-cell patch-clamp recording configuration had been obtained, cell superfusion was via a home-built rapid solution exchange (< 1 s) device, which was used to change superfusate. All superfusates were maintained at 35–37 °C.

All solutions were made with deionised Milli-Q water (Millipore Systems). K_2_-creatine phosphate was purchased from Merck Chemicals Ltd, flufenamic acid from Tocris, and all other chemicals from Sigma-Aldrich unless otherwise stated. 100 mM GdCl_3_, LaCl_3_, ruthenium red, and 1 M amiloride-HCl were made up in H_2_O and 100 mM flufenamic acid in DMSO as stock solutions which were kept at − 20 °C.

### 2.3 Estimation of single channel conductance through “noise analysis”

Single channel currents were estimated from the variance of the Na^+^-dependent background current, calculated from the integral of the power spectral density. The single channel conductance was calculated as the slope of the single channel current-voltage relation between − 110 and − 80 mV, where the current-voltage relation approached the asymptote predicted by the Goldman-Hodgkin-Katz (GHK) flux equation (see below). Further details are provided in the online supplementary information and have been described elsewhere [30].

### 2.4 Data analysis

Whole cell current analysis was performed using Clampfit from the pClamp 10.0 software suite. Statistical analysis was performed using Microsoft Office Excel (Microsoft Corporation), Origin (OriginLab Corporation) and Prism (Graphpad Software, Inc.). Graphs were drawn using Graphpad Prism or Igor Pro (Wavemetrics Inc.). All data are expressed as mean ± SEM.

### 2.5 Computer modelling of AVN activity

**Fig. 1:**
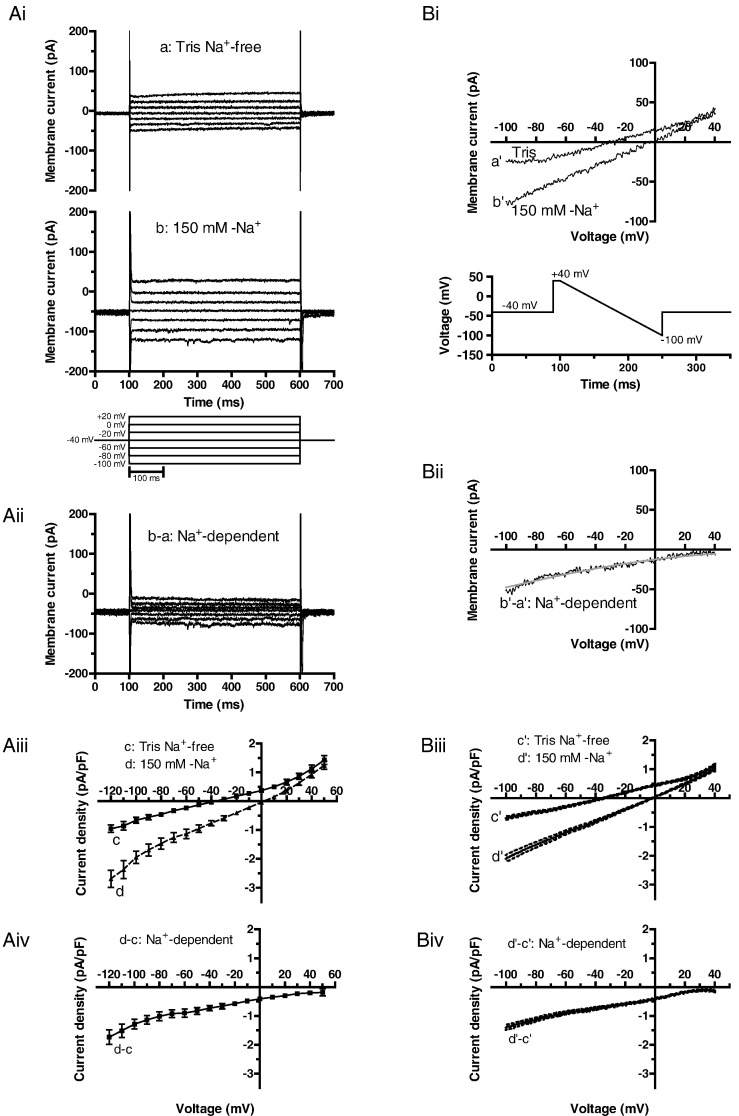
Background currents elicited by voltage step (Ai–Aiv) and descending voltage ramp (Bi–Biv) protocols in rabbit AVN cells. Ai: Representative families of currents in Tris Na^+^-free and 150 mM-Na^+^ solutions. For clarity of display, only selected current traces are shown (protocol is shown underneath). Aii: Representative Na^+^-dependent inward background currents obtained by subtracting the currents in Tris Na^+^-free from those in 150 mM-Na^+^ solution (see Ai). Aiii: Mean current-voltage relations for currents (end pulse) in Tris Na^+^-free and 150 mM-Na^+^ solutions (mean ± SEM, n = 8 cells). Aiv: Mean current-voltage relation for the subtracted Na^+^-dependent inward background current, I_B,Na_ (mean ± SEM, n = 8 cells). Bi: Representative currents respectively in Tris Na^+^-free and 150 mmol/L-Na^+^ solutions (protocol is shown underneath). Bii: Representative Na^+^-dependent inward background current obtained by subtracting the current in Tris Na^+^-free from that in 150 mM-Na^+^ solution (see Bi); grey line denotes a fit to the data with a Goldman-Hodgkin-Katz (GHK) current equation for diffusion of permeant ions. Biii: Mean current-voltage relations for currents in Tris Na^+^-free and 150 mM-Na^+^ solutions (mean ± SEM (dotted lines), n = 61 cells). Biv: Mean current-voltage relation for the subtracted Na^+^-dependent inward background current, I_B,Na_ (mean ± SEM, n = 61cells).

The most biophysically detailed available cell and tissue models of the AVN are those for rabbit AVN by Inada et al [7]*.* Only the ‘N’ cell model exhibits automaticity [7] and this was therefore used to investigate the influence of *I*_B,Na_ on rabbit AVN cell spontaneous and driven action potentials. The Goldman-Hodgkin-Katz (GHK) flux equation was chosen to simulate I_B,Na_:

(1) $$I_{B,\mathrm{Na}}=P_{\mathrm{Na}}V_{m}\left(\frac{F^{2}}{\mathrm{RT}}\right)\left(\frac{{\left[Na^{+}\right]}_{i}-{\left[Na^{+}\right]}_{o}\exp\left(-\frac{V_{m}F}{\mathrm{RT}}\right)}{1-\exp\left(-\frac{V_{m}F}{\mathrm{RT}}\right)}\right)$$

where *P*_*Na*_ is the Na^+^ permeability, *V*_*m*_ is the membrane potential, *F* is Faraday's constant, *R* is the gas constant, T is the absolute temperature, and [Na^+^]_*i*_ and [Na^+^]_*o*_ are the intracellular and extracellular Na^+^ concentrations. *P*_*Na*_ was determined by fitting I_B,Na_ from Fig. 1Biv by the GHK flux equation (P_Na_ = 7.308 × 10^− 1^ L/s; cell capacitance, C_m_ = 29 pF [7], [31]). To eliminate I_B,Na_ from the AV node, I_B,Na_ calculated as above (but for physiological [Na^+^]_*i*_ and [Na^+^]_*o*_) was subtracted from the N cell model. The conduction velocity was determined using a 1D string model. The string model consisted of 100 elements (myocytes). The length of each element was 100 μm. Conduction was calculated using the reaction-diffusion equation:

(2) $$C_{m}\frac{\partial V_{m}}{\partial t}=\nabla \cdot D\nabla V_{m}-I_{\mathrm{ion}}+I_{\mathrm{stim}}$$

where *D* is the diffusion coefficient, *I*_*ion*_ is the ionic current and *I*_*stim*_ is the stimulation current. *D* was taken to be 0.003 mSmm^2^ (equivalent to a coupling conductance of 0.3 mS). The stimulus was applied at the first three elements. The conduction velocity was determined as the average conduction velocity calculated from the 30^th^ element to the 70^th^ element.

## 3 Results

### 3.1 Background current during voltage steps and ramps

**Fig. 2:**
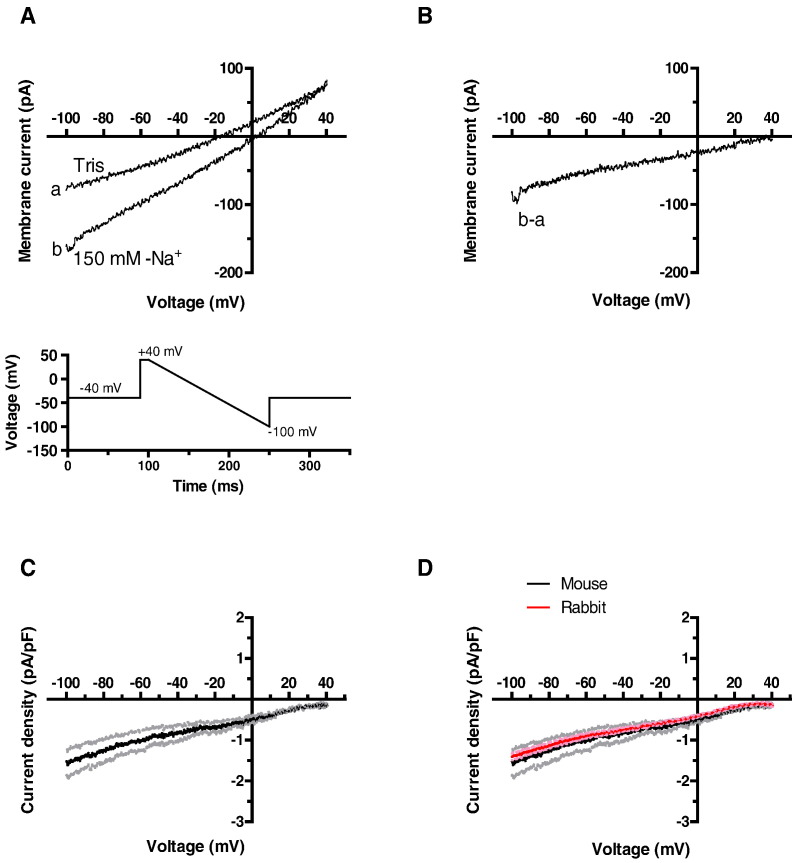
Na^+^-dependent inward background current (I_B,Na_) in mouse AVN cells. A: Representative net currents in Tris- (a) and Na^+^ (b) -containing solutions elicited by descending voltage ramp (lower panel). B: The resulting Na^+^-sensitive subtraction current I_B,Na_ (b–a). C: The mean I–V relation for I_B,Na_ from mouse AVN cells (mean ± SEM (dotted lines), n = 6 cells). D: Overlay of the mean Na^+^-dependent background currents I_B,Na_ from mouse (black line and grey dotted lines show mean ± SEM; n = 6 cells) and rabbit (red line and pink dotted lines show mean ± SEM; n = 61 cells), indicating the similarity between I_B,Na_ obtained from the two species.

Net background current and Na–Tris difference current were studied using voltage step and ramp protocols (lower panels in Fig. 1Ai and Bi). In the presence of 150 mM extracellular Na^+^, voltage steps to potentials between − 120 and + 50 mV (in 10 mV increments, pulse frequency 0.2 Hz) elicited currents that showed little time-dependence during the applied voltage command. Holding current at − 40 mV was inward under these conditions (Fig. 1Ai panel b). When the superfusate was Tris-free, both outward and inward current components were smaller (Fig. 1Ai panel a) and the holding current became markedly less inward. Representative Na^+^–Tris difference currents are shown in Fig. 1Aii and were time-independent and inwardly directed over the full range of membrane potentials tested. Mean current-voltage (I–V) relations for net current in Na^+^- and Tris-containing solutions are shown in Fig. 1Aiii, whilst the mean I–V relation for Na^+^-sensitive (Na^+^–Tris difference) current is shown in Fig. 1Aiv, and was inwardly directed across the entire range of test potentials. The time-independence of the currents observed during voltage steps enables the use of a voltage-ramp protocol to survey background current rapidly across a wide range of potentials. Thus, we also examined currents elicited by a descending ramp protocol (between + 40 and − 100 mV over 150 ms; frequency 0.2 Hz). Representative currents in Na^+^-containing and Tris-containing solutions are shown in Fig. 1Bi, with the corresponding Na^+^–Tris difference current shown in Fig. 1Bii. The net current in Na^+^-containing solution was linear, reversing close to 0 mV (Fig. 1Bi), whilst the Na^+^-dependent (Na^+^–Tris difference) current was inwardly directed across the entire potential range of the voltage ramp. Mean I–V relations for net current in Na^+^ and Tris-containing solutions are shown in Fig. 1Biii, whilst mean Na^+^-sensitive difference current is shown in Fig. 1Biv. The mean I–V relation for Na^+^-sensitive difference current during voltage-ramps was similar to that for currents elicited by voltage steps (compare Fig. 1Aiv and Biv); consequently the voltage ramp protocol was employed for most subsequent experiments. The presence of a Na^+^-sensitive inward background current was not exclusive to rabbit AVN, as we also recorded a similar current from murine AVN cells (Fig. 2). Fig. 2A shows representative mouse AVN cell currents in Na- and Tris- containing solutions, elicited by the same voltage ramp protocol as used to record rabbit AVN cell I_B,Na_. Fig. 2B shows the Na^+^–Tris difference current, representing I_B,Na_, whilst Fig. 2C shows mean murine AVN cell I_B,Na_ from 6 experiments. Fig. 2D shows the mean current-voltage (I–V) relation for I_B,Na_ from murine AVN cells, with the mean current from rabbit cells superimposed in red. The I–V relations for I_B,Na_ for the two species were similar, indicating both that the current is not restricted to rabbit AVN and that it was remarkably similar in amplitude in mouse and rabbit AVN cells.

### 3.2 Na^+^ dependence and effects of ionic substitution

**Fig. 3:**
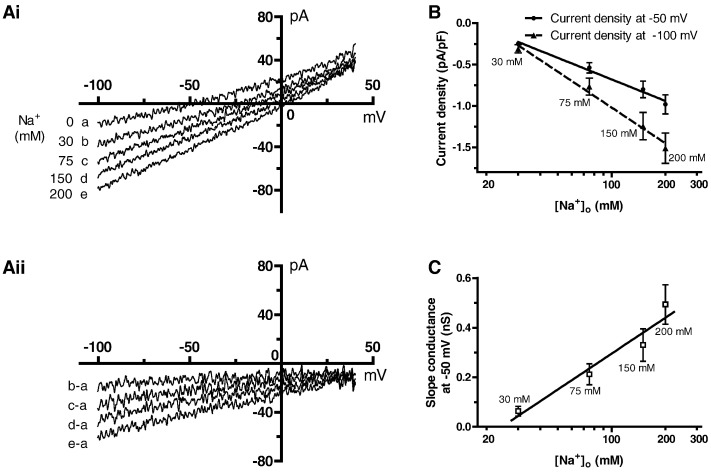
Background inward current depends on the concentration of Na^+^ in the extracellular solution ([Na^+^]_o_). Ai: Representative currents in various Na^+^ concentrations (recorded from a same cell). Aii: The difference curve b-a is the Na^+^-dependent current in 30 mM Na^+^; c-a, 75 mM Na^+^; d-a, 150 mM Na^+^; and e-a in 200 mM Na^+^ solution. B: the relations between log [Na^+^]_o_ and the current densities of the Na^+^-dependent currents at − 50 and − 100 mV in various [Na^+^]_o_ (mean ± SEM, n = 8 cells). C: The relation between log[Na^+^]_o_ and the slope conductance of the Na^+^-dependent currents at − 50 mV in various [Na^+^]_o_ (mean ± SEM, n = 8 cells). The straight black and dashed lines in B and C show the linear relations.

The effects of altering [Na^+^]_o_ between 0 and 200 mM on net current magnitude and profile are shown in Fig. 3Ai: as [Na^+^]_o_ was progressively reduced from 150 mM, the net inward current component became smaller and the current reversed at progressively more negative voltages. Fig. 3Aii shows (for the same cell as Fig. 3Ai) Na^+^-sensitive difference currents in 30, 75, 150 and 200 mM [Na^+^]_o_. Fig. 3B shows the concentration-dependence of the [Na^+^]_o_-dependent current at two selected voltages (− 50 and − 100 mV), showing a linear dependence of current density on log [Na^+^]_o_, whilst Fig. 3C shows the [Na^+^]_o_-dependence of the slope conductance of the Na^+^-sensitive current. The linear dependence of current magnitude on log [Na^+^]_o_ is similar to that reported for SAN I_B,Na_ [19]. The mean slope conductance at − 50 mV in Tris-containing [0 Na^+^] solution was 0.54 ± 0.03 nS (n = 61; compared to 0.45 ± 0.18 nS previously reported for SAN cells under similar conditions [19]) whilst in 150 mM mean slope conductance increased to 0.91 ± 0.05 nS (compared to 0.87 ± 0.33 nS for SAN cells [19]). Considered collectively, the data in Fig. 3 demonstrate a strong dependence of current magnitude on [Na^+^]_o_ and throughout the rest of this report this current component is denoted I_B,Na_.

**Fig. 4:**
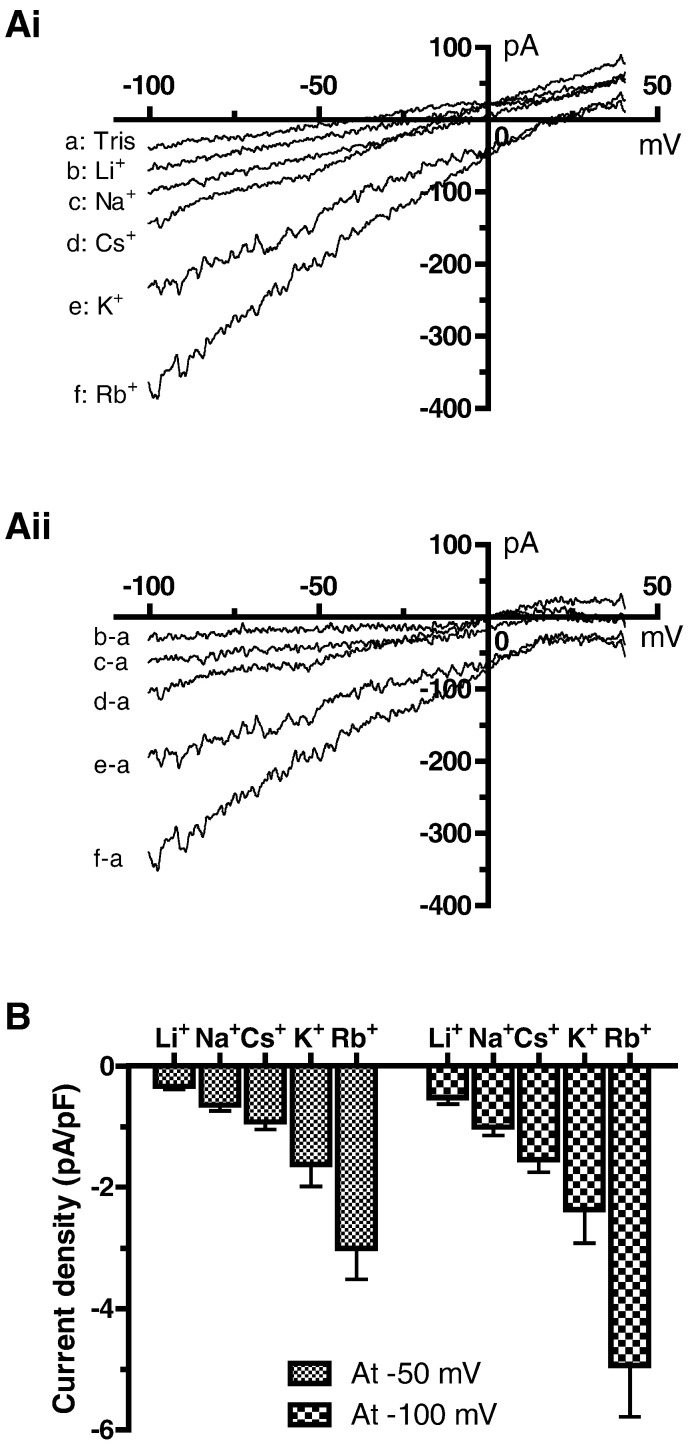
Background inward current with differing external monovalent cations. Ai: Representative current traces in various monovalent cation external solutions as indicated (recorded from the same cell). The background inward current amplitude increased in the order of Li^+^ < Na^+^ < Cs^+^ < K^+^ < Rb^+^. Aii: The difference curves between Tris and various monovalent cation external solutions. B: Mean background inward current densities at − 50 and − 100 mV in various monovalent cation external solutions (mean ± SEM, n = 7 cells), indicating the channel mediating this background current exhibits poor cation selectivity.

Fig. 4 shows the effects of monovalent cation substitution on the profile and magnitude of the background current. Fig. 4Ai shows records from a single cell in solutions containing 150 mM of Tris, Li^+^, Na^+^, Cs^+^, K^+^ and Rb^+^, whilst Fig. 4Aii shows Tris-difference currents for each metal cation. Fig. 4B shows mean current density plots for current at − 50 and − 100 mV for I_B,Na_ and the equivalent current with the other cations. The current was monovalent non-selective, with its amplitude largest in Rb^+^ and smallest in Li^+^ (Rb^+^ > K^+^ > Cs^+^ > Na^+^ > Li^+^). The estimated relative slope conductance ratios at − 50 mV for these ions compared with Na^+^ were, respectively, 5.96, 3.00, 2.33, 1.00 and 0.58. For the SAN, Hagiwara et al used the relative slope conductance ratio for K^+^ compared to Na^+^ from similar experiments to estimate the reversal potential (*E*_rev_) for the net monovalent NSCC, assuming physiological [Na^+^]_o_ and [K^+^]_i_ concentrations; this yielded a value of “around − 21 mV” [19]. Using a *P*_K_/*P*_Na_ ratio of 3.0 from the present study, together with [Na^+^]_o_ of 140 mmol/L, [Na^+^]_i_ of 8 mM and [K^+^]_o_ of 5.4 mM, [K^+^]_i_ of 140 mM, we obtained an E_rev_ of − 26.9 mV, which is close to the value estimated by Hagiwara et al for SAN cells [19]. As an additional check, we used the above ion concentrations together with a *P*_K_/*P*_Na_ value for SAN cells of 2.27 from [19], and closely matched the previously estimated E_rev_ for SAN cells, with a derived value of − 20.3 mV.

### 3.3 Sensitivity of I_B,Na_ to pharmacological inhibition

**Fig. 5:**
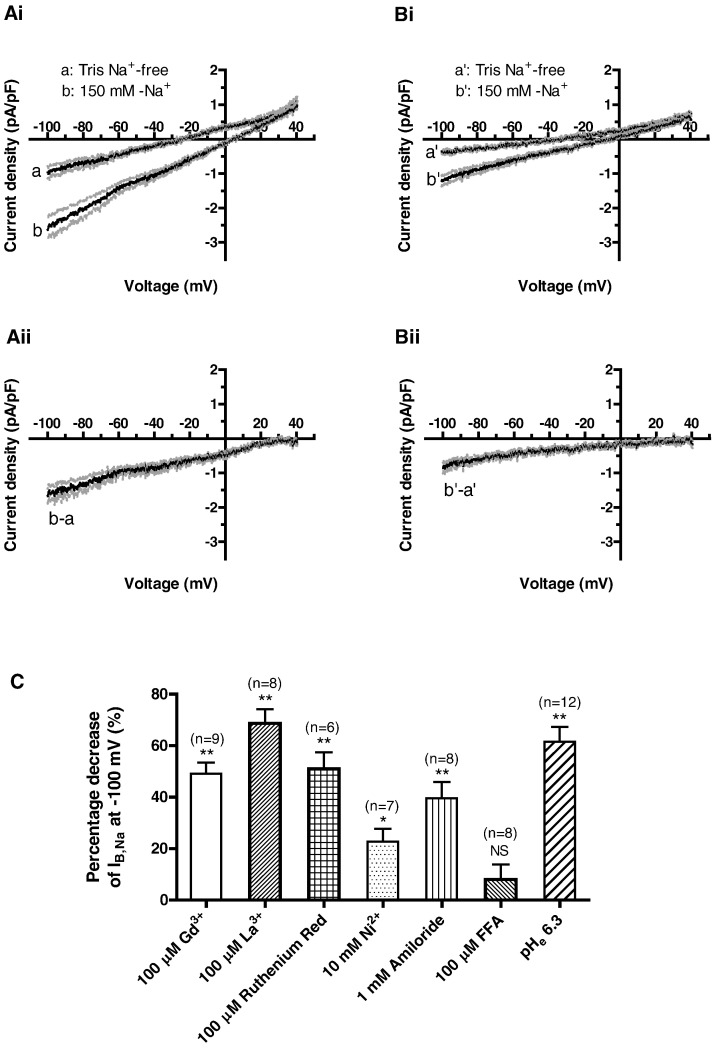
Effects of gadolinium (Gd^3 +^), lanthanum (La^3 +^), ruthenium red, nickel (Ni^2 +^), amiloride, flufenamic acidic (FFA) and acidic pH on Na^+^-dependent inward background current (I_B,Na_). Ai and Aii: in control condition, mean I–V relations for currents in Tris Na^+^-free and 150 mM-Na^+^ solutions (Ai), and mean I_B,Na_ (Aii) (mean ± SEM (dotted lines), n = 9). Bi and Bii: with application of 100 μM Gd^3 +^, mean I–V relations for currents in Tris Na^+^-free and 150 mM-Na^+^ solutions (Bi), and mean I_B,Na_ (Bii) (mean ± SEM (dotted lines), n = 9). C: A summary of the effects of 100 μM Gd^3 +^, 100 μM La^3 +^, 100 μM ruthenium red, 10 mM Ni^2 +^, 1 mM amiloride, 100 μM flufenamic acid (FFA), and acidosis of pH 6.3 on I_B,Na_ at − 100 mV. *P < 0.05, **P < 0.01; the numbers of cells for each experiment are given in parentheses.

Taken together, the data in Fig. 1, Fig. 2, Fig. 3, Fig. 4 suggest that I_B,Na_ is the [Na^+^]_o_-sensitive component of a background monovalent non-selective cation channel (NSCC) current. Gd^3 +^ ions block a number of NSCCs [32] and consequently we tested the effects of 100 μM Gd^3 +^ on I_B,Na_. Fig. 5Ai and Aii show mean currents in Na^+^-containing and Tris-containing solution, whilst Fig. 5Bi and Bii show comparable data for the same sample of cells, when the superfusate contained 100 μM Gd^3 +^. As shown in Fig. 5Aii and Bii, Gd^3 +^ led to a reduction in I_B,Na_ amplitude across the tested range. In 9 cells, at − 100 mV I_B,Na_ amplitude was decreased by 49.1 ± 4.3% by this concentration of Gd^3 +^ (Fig. 5C). In a further 9 cells, 1 μM Gd^3 +^ inhibited I_B,Na_ by 52.4 ± 9.2%. A second lanthanide, lanthanum (La^3 +^) also inhibited I_B,Na_, with 100 μM La^3 +^ blocking the current by 68.6 ± 5.5% (n = 8; Fig. 5C). 1 mM La^3 +^ inhibited I_B,Na_ by 71.6 ± 3.9% (n = 8). Ruthenium red (100 μM), which inhibits multiple cation channels [32], [33], inhibited I_B,Na_ by 50.9 ± 6.5% (n = 6; Fig. 5C). By contrast, increasing the [Ni^2 +^] in the superfusate from 2 to 10 mM (a concentration sufficient to inhibit maximally cardiac Na–Ca exchange [34]) reduced I_B,Na_ by only ~ 20% (Fig. 5C). Amiloride has been suggested to inhibit partially I_B,Na_ [19] in the SAN and we found it to inhibit AVN I_B,Na_ by ~ 40% (Fig. 5C). Flufenamic acid (FFA) has been shown to inhibit TRMP4-related NSCCs in SAN cells [35]; however it was without significant effect on I_B,Na_ (Fig. 5C). By contrast, lowering the pH of the superfusate from 7.4 to 6.3 inhibited the current by > 60% (Fig. 5C). Murine AVN cell I_B,Na_ was also reduced by acidic pH_e_ (data not shown).

### 3.4 Estimating single channel conductance for I_B,Na_

**Fig. 6:**
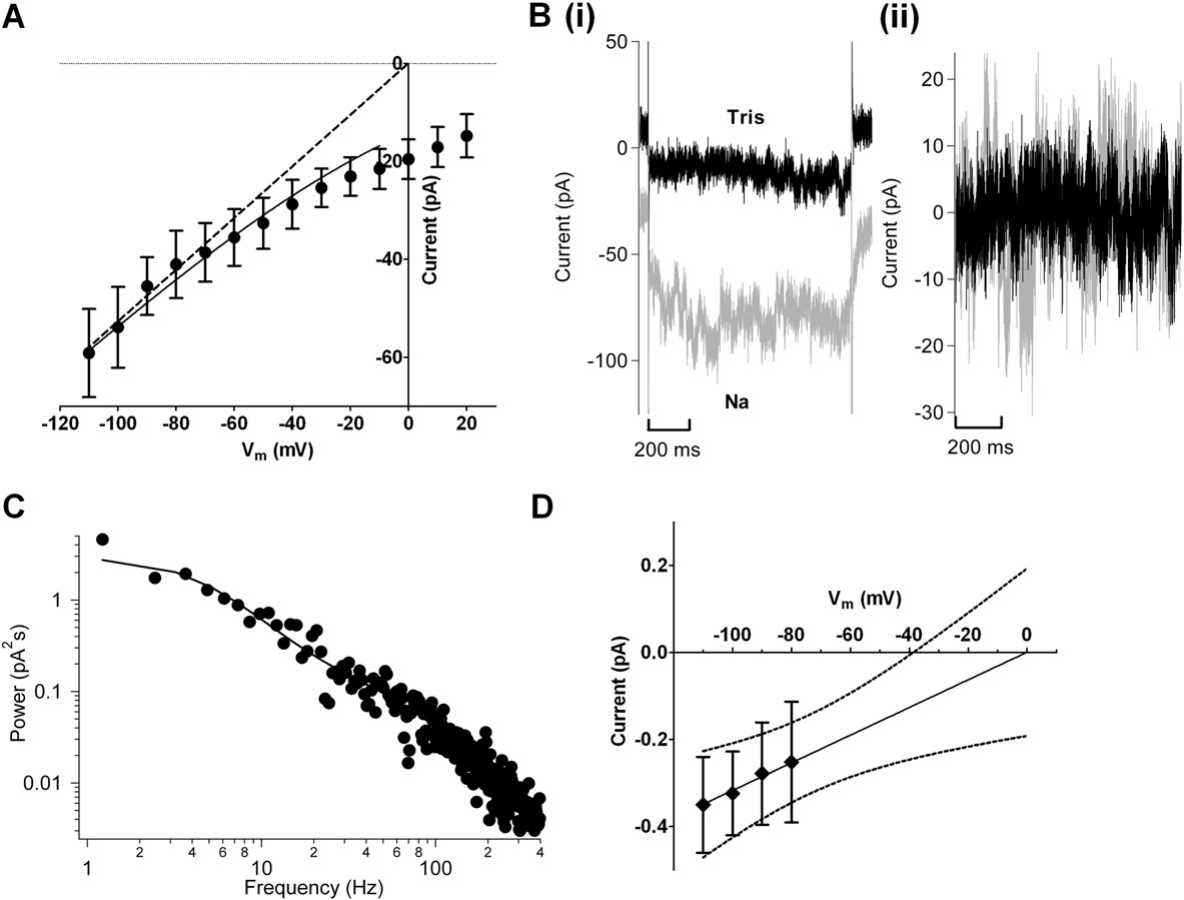
The single-channel conductance of I_B,Na_ estimated from power spectral analysis. (A) Mean whole cell Na-dependent inward current-voltage relations from 6 AVN cells. Currents were recorded during voltage steps ranging from − 110 to + 20 mV. Currents recorded in Tris-based solution were subtracted from currents recorded in Na-based solution. Solid line represents a fit to Eq. (1) (full data range not fitted because the equation becomes indeterminate at 0 mV). Dashed line indicates the asymptotic current-voltage relation for the unidirectional flux converging on an E_rev_ of 0 mV. (Bi) Example current traces recorded in Na (grey) and Tris (black) -based solutions on stepping to − 100 mV. (Bii) Example DC-subtracted current traces recorded at − 100 mV in Na (grey) and Tris (black)-based solutions. Data correspond to those shown in (i). (C) Example power spectral density. Data are from the cell shown in B. Solid line represents a fit to equation S1 (see online Supplementary information). D Mean unitary background channel Na current-voltage relations at the asymptote. Unitary current amplitudes were calculated from the integral of the power spectral density at each voltage according to Eq. (S2). Data correspond to the 6 cells shown in ‘A’. Solid line was fitted by linear regression constrained to reverse at 0 mV. The slope gives a mean open channel conductance of 3.2 ± 1.2 pS. Dotted lines show the 95% confidence intervals.

To our knowledge, at present no data are available regarding the single channel conductance of I_B,Na_ channels for any cardiac cell type. In principle, the difference in power spectra of the current “noise” between Na^+^-containing and Tris-containing (Na^+^-free) external solutions can be used to estimate single channel conductance, because the whole-cell current variance is a function of the current amplitudes through single open channels, and consequently the power spectra at any voltage provide a measure of the unitary current amplitude at that voltage [36]. We used currents generated by voltage step commands between − 110 and + 20 mV to obtain the Na^+^-dependent (Na^+^-Tris difference) current, deriving from their current-voltage relation the asymptote shown in Fig. 6A. Over the voltage range at which the asymptote was achieved (− 110 to − 80 mV inclusive), the DC component of current in both Na^+^-containing and Tris-containing solutions was removed (Fig. 6Bi and Bii), and the power spectra calculated. The power spectrum of the Na^+^-dependent current, calculated as the difference between the power spectrum in Na^+^-containing and Na^+^-free solutions, was fitted with equation S1 (Fig. 6C). The power spectra at each voltage were integrated to obtain the variance, from which the unitary current amplitudes were estimated (Fig. 6D). The slope conductance of the mean unitary current voltage–relation was 3.2 ± 1.2 pS.

### 3.5 Investigating the potential physiological role of I_B,Na_

**Fig. 7:**
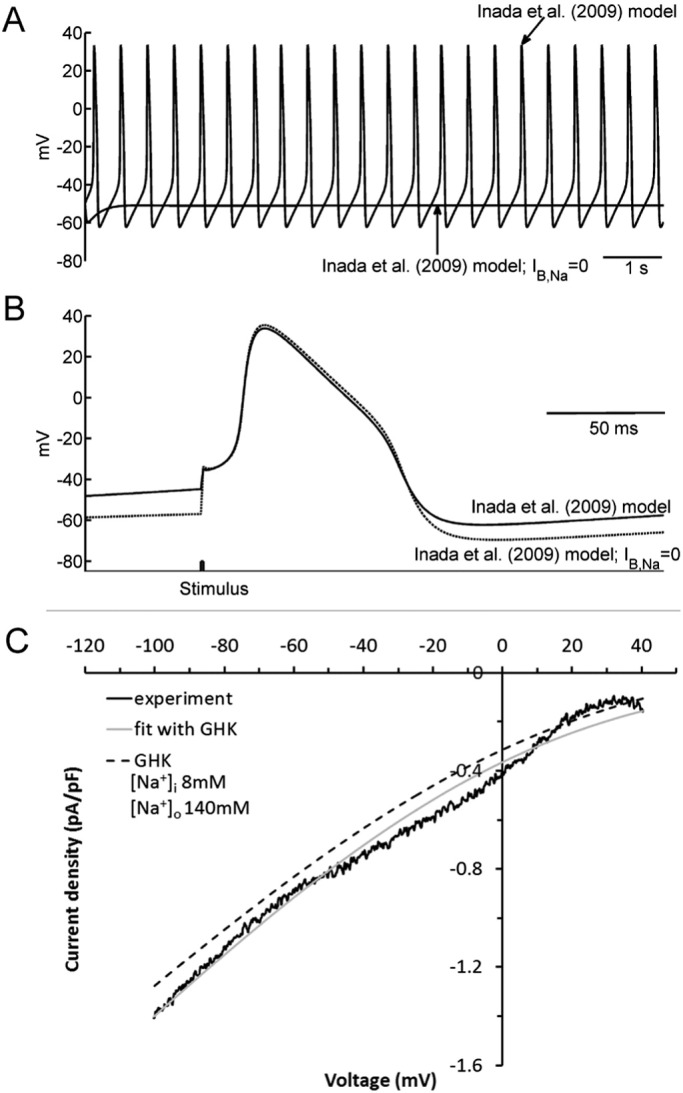
Predicted role of I_B,Na_ in the AV node action potential. (A) predicted role of I_B,Na_ in AV node pacemaking. The traces show electrical activity calculated using the N cell model from Inada et al. [7] before and after the elimination of I_B,Na_ from the N cell model. In the presence of I_B,Na_, the model shows robust pacemaking, but after elimination of I_B,Na_ pacemaking is abolished. (B) Predicted role of I_B,Na_ in the driven AV node action potential. The 10th action potential during 2.5 Hz stimulation is shown. Action potentials before and after the elimination of I_B,Na_ are shown. After the elimination of I_B,Na_, the resting membrane is hyperpolarized. (C) Current-voltage relationships for I_B,Na_. Solid black line, experimental I_B,Na_ from Fig. 1Biv. Solid grey line, GHK flux equation fitted to experimental data. Dashed black line, current-voltage relationship predicted by the GHK equation under physiological conditions (for all simulations [Na^+^]_*i*_ = 8 μM; [Na^+^]_o_ = 140 mM; intracellular and extracellular [K^+^] were set, respectively to 140 mM and 5.4 mM). As shown in panel C (dashed line), the GHK simulated current under ‘physiological’ conditions was slightly smaller than that recorded experimentally (with 150 mM [Na^+^]_o_ and 0 [Na^+^]_i_). It is the smaller current under ‘physiological’ conditions that was incorporated into action potential simulations.

None of the agents tested in the experiments described in Fig. 5 produced complete inhibition of I_B,Na_, nor would they be expected to be I_B,Na_-selective under action potential (AP) recording conditions. Therefore we reasoned that, in the absence of a specific blocker, the potential role of I_B,Na_ in electrical activity of the AVN may best be investigated using computer modelling. The “N” cell model from the Inada et al. AVN electrophysiology model, which exhibits spontaneous activity in the absence of external stimulation [7] was therefore chosen to study the influence of I_B,Na_. Fig. 7A shows spontaneous APs produced by this cellular model. It contains background current (which can be interpreted as the sum of all background currents) and the effect of block of I_B,Na_ was simulated by subtracting I_B,Na_ calculated using the GHK flux equation (Eq. (1)) fitted to experimental data (Fig. 1Biv). After block of I_B,Na_, pacemaking ceased, because (consistent with the block of an inward current) during the pacemaker potential the membrane potential now failed to reach the threshold potential, attaining quiescence at a value of ~− 53 mV. Experimental data indicate that AVN cells exhibit ‘zero current’ potentials of ~− 40 mV (e.g. [6], [20], [21], [22]) and additional simulations were performed (online Supplement Fig. S1) in which L-type Ca current was abolished to induce quiescence in the presence of I_B,Na_. This intervention induced quiescence at − 40 mV; thus, the effect of I_B,Na_ removal in Fig. 7A was to produce a hyperpolarization of ‘resting’ potential. Under normal conditions with SAN dominance, the AVN is driven and does not show pacemaking. Fig. 7B shows the effect of block of I_B,Na_ on the driven AP. The AP shape and duration were not affected, but the resting membrane was hyperpolarized (again consistent with the block of an inward current; Fig. 7B). Hyperpolarization of the resting membrane may affect excitability and, therefore, conduction velocity; this was examined using a 1D string model (see Methods). The conduction velocity obtained under control conditions (I_B,Na_ present; 16.7 cm s^− 1^) is typical of the rabbit AVN [7]. Block of I_B,Na_ decreased the conduction velocity by ~ 20% (to 13.3 cm s^− 1^). Fig. 7C shows that experimental data for I_B,Na_ were well fitted by Eq. (1). Additionally, the predicted I–V relation for I_B,Na_ under ‘physiological’ conditions ([Na]_o_ set to 140 mM; [Na]_i_ set to 8 mM) was inward across the entire range of physiologically relevant membrane potentials (Fig. 7C).

## 4 Discussion

This study provides the first evidence for the presence and role of I_B,Na_ in the AVN and, to our knowledge, the first experimental estimate for the single channel conductance of the channels that underlie I_B,Na_ for any cardiac cell type.

### 4.1 On the nature of I_B,Na_

The current-voltage relation for net background current under bivalent conditions (Na^+^ outside/Cs^+^ inside) in this study was linear, reversing close to 0 mV, consistent with a dominant identity of total background current under our recording conditions as a NSCC. The estimated E_rev_ for this monovalent NSCC, with physiological Na^+^ and K^+^ values, of − 26.9 mV indicates that, as previously suggested for the SAN [19], it would carry inward current over the diastolic potential range in AVN cells. I_B,Na_ was measured as the external Na^+^-sensitive component of this NSCC, under the same conditions as used previously to study an analogous conductance in SAN cells [19]. Our results indicate that I_B,Na_ is both present in the AVN and also of similar magnitude to that reported for the SAN [19]. The strong similarity between I_B,Na_ in rabbit and murine AVN cells seen here suggests conservation of the current in cells from this region across species. There is prior evidence for the presence of an I_B,Na_ in non-pacemaker cells, but one that it is of substantially smaller magnitude [19], [37].

The GHK voltage dependence and Eisenmann III permeability sequence for I_B,Na_ distinguish this current from voltage-dependent ‘persistent’ or ‘late’ Na current [38]. Additionally, although it has been suggested that minor transport modes of Na–Ca exchange might account for cardiac background inward current [25], [39] and AVN cells exhibit a robust Na–Ca exchange current [26], [40], the persistence in our experiments of I_B·Na_ in the presence of a maximally effective Na–Ca exchange blocking concentration of Ni^2 +^ [34], together with the measured cation permeability sequence and results of “noise” analysis, argue against a significant contribution of Na–Ca exchange to I_B,Na_. Similarly, the presence of I_B,Na_ in the absence of external Ca^2 +^, together with its low single channel conductance and insensitivity to FFA distinguish this current from the TRPM4-mediated FFA-sensitive, Ca-activated NSCC observed for SAN cells [35]. Furthermore, despite some sensitivity to lanthanides and other manoeuvres that inhibit NSCCs, the permeability sequence and low single channel conductance for I_B,Na_ seem difficult to reconcile with properties of other members of the transient receptor potential (TRP) family of NSCCs [32]. For example, whilst TRPV4 has an Eisenmann IV permeability sequence for monovalent cations, close to the sequence for I_B,Na_, and is also sensitive to ruthenium red [41], its single channel conductance at negative voltages lies between 30 and 60 pS [42], 10-fold or more our estimate for channels mediating I_B,Na_. On the other hand, the single channel conductance estimated here is close to that for the epithelial Na channel (ENaC; 4–5 pS), but ENaC has a higher sensitivity to inhibition by amiloride and in contrast with I_B,Na_ has a high Na/K relative permeability [43]. Low conductance NSCC behaviour has been induced in the Na/K pump by exposure to the marine toxin, palytoxin (PTX), with a PTX induced single channel conductance of ~ 7 pS [24]. The P_K_/P_Na_, P_Cs_/P_Na_, and P_Rb_/P_Na_ ratios for this toxin-induced NSCC were reported to be 1.13, 1.01 and 1.11 [24], unlike the relative conductance ratios for AVN I_B,Na_ respectively of 2.33, 3.00 and 5.96. Although the molecular architecture of a number of ion channel transcripts in the AVN has been mapped, at present this information does not extend to NSCC candidates [44], [45].

### 4.2 Physiological role of I_B,Na_

The lack of an identified molecular correlate for channels carrying I_B,Na_ precludes elucidation of its physiological role(s) through genetic modification and no selective pharmacological inhibitor of the current has yet been discovered. Additionally, Na substitution cannot be used under physiological recording conditions to discriminate I_B,Na_ from other conductances as this intervention would also affect I_f_ and Na–Ca exchange current. Computational modelling thus affords the only available means of assessing the physiological contribution of I_B,Na_. One study has suggested that I_f_ and I_B,Na_ may play ‘reciprocal’ roles in pacemaking of SAN cells, in which membrane hyperpolarisation following a reduction in either current leads to augmentation of the other, thereby stabilising pacemaker rate [46]. In another modelling study I_B,Na_ contributed ~ twice the background inward current to I_NCX_ during SAN pacemaking [47]. A third simulation study suggested a 30% decrease in spontaneous rate of central SAN cells following I_B,Na_ inhibition when I_f_ was present, with a greater effect when I_f_ was blocked [48]. Whilst there may be quantitative differences between studies, simulation evidence supports the notion that I_B,Na_ can influence spontaneous activity of the SAN.

Similar to the SAN, cells from the AVN also lack appreciable I_K1_ at diastolic potentials and exhibit a high membrane resistance, which makes membrane potential labile over the diastolic potential range [21], [31], [49], [50]. The data from our simulations are consistent with a significant role for I_B,Na_ in both pacemaker and conduction properties of the AVN. In the spontaneously active ‘N’ cell model [7], in the absence of I_B,Na_ membrane potential failed to reach the threshold for AP initiation, leading to an arrest of spontaneous activity. When APs were triggered, removal of I_B,Na_ did not alter AP shape, but hyperpolarized membrane potential, associated with a decreased excitability manifested as a (20%) slowed conduction velocity. I_B,Na_ may also have pathophysiological significance in the AVN: in previous experimental studies, extracellular acidosis reduced both spontaneous rate and net background current of single cells (time-independent current with voltage gated L-type Ca^2 +^ and rapid delayed rectifier (I_Kr_) K currents inhibited, with ‘physiological’ recording solutions) [26] and it also slowed AVN conduction [51]. The sensitivity of AVN I_B,Na_ to external pH demonstrated here may, at least in part, contribute to these earlier observations. We tested this idea by incorporating partial I_B,Na_ reduction (of 60%) to mimic consequences of effects of pH 6.3 on this current and in consequence spontaneous rate was reduced by ~ 36% (see Supplemental Fig. S2); with concomitant I_Ca,L_ and I_Kr_ reduction this reduction was increased to ~ 53% (see Supplemental Fig. S2). These results are consistent with partial I_B,Na_ reduction (alone and synergistically with additional channel effects) being able to contribute to (patho)physiological modulation of AVN cell spontaneous rate.

The smaller amplitude of I_B,Na_ in non-pacemaker cell types [19], [37] together with the concomitant presence of current generated by channels for inwardly rectifying K^+^ current, I_K1_, likely limits the impact of this current on electrogenesis in those cells, though it is possible that the current may still influence Na homeostasis [52].

### 4.3 Limitations

Although to our knowledge this is the first study to provide an estimate of single channel conductance for channels mediating cardiac I_B,Na_, “noise analysis” is an indirect rather than direct method of observing single channel activity. Its application in the present study is predicated on the assumption that Na^+^ removal affected only background current. This is a reasonable assumption given that the experimental solutions utilized in this study (and the earlier report of SAN I_B,Na_ [19]) were designed to inhibit major overlapping ion channel and transporter currents. For example, activation of cardiac Na-dependent K^+^ channels requires ~ 20 mM intracellular [Na^+^] [53], [54] and, our pipette solution was both largely Cs^+^-based and Na^+^-free, which precludes K_Na_ current activation in our experiments. Additionally, the presence of strophanthidin and nickel in the external solution makes significant contamination by Na-K-ATPase or Na/Ca exchange currents unlikely; moreover, the properties of the power spectrum obtained indicate that the currents were predominantly produced by channels showing gating behaviour, ruling out transporter-currents. Thus, it is reasonable to conclude that the channels identified through power-spectral analysis of Na^+^-Tris difference currents are distinct. Estimation of single channel conductance through this method required measurements in the voltage range − 80 to − 110 mV, rather than at diastolic potentials, in order to obtain currents of adequate size for power spectral analysis. This range of voltages was chosen as it avoided the underestimation of conductance through the effects of GHK rectification (i.e. the current voltage relation of the Na-dependent current achieved the asymptote in this voltage range). Future work to obtain direct measurements of single I_B,Na_ channels would provide valuable independent validation of the single channel conductance estimate obtained from our analysis. However, the low single channel conductance may make such measurements somewhat challenging to make.

The AVN is electrically and structurally heterogeneous [2], [55]. Whilst isolated AVN cell populations are also heterogeneous [31], [56], [57], it is not possible to attribute a precise origin from within the AVN to cells studied; thus the present study does not address directly issues of potential regional differences in the distribution within the AVN of I_B,Na._ The fact that the underlying genetic basis for the channel (in any cardiac region) remains to be determined also precludes mapping I_B,Na_ channel transcript or protein levels within AVN sub-regions. In principle, this limitation also applies to prior I_B,Na_ data on the SAN [19]. However, (i) the observation that I_B,Na_ was recorded from a large number of rabbit AVN cells with relatively small variation (Fig. 1Aiv and Biv), and (ii) the striking concordance between the mean I_B,Na_ magnitude in rabbit and murine AVN cells (Fig. 2D) are consistent with homogeneous distribution of I_B,Na_ in the AVN and suggest that this potential limitation is unlikely to detract from the main conclusions and implications of this study. The lack of a selective pharmacological inhibitor for I_B,Na_ means that it is not currently possible to validate our AP simulation results experimentally. This limitation is shared with any experimental study of I_B,Na_.

A previous study suggested that I_f_ and I_B,Na_ may play ‘reciprocal’ roles in stabilising pacemaking rate of SAN cells, in which membrane hyperpolarisation following a reduction in either current leads to augmentation of the other [46]. Quiescence rather than stabilization of pacemaking was observed in the present study of AVN cells when I_B,Na_ was removed from the model. A prior experimental study in which I_f_ was compared between rabbit SAN and AVN cells found the current to be smaller in the latter [58] and so it is possible that the relative roles of I_f_/I_B,Na_ differ in the two cell types. However, the relative roles of individual currents in a given model depend on model paramaterization and we cannot rule out that quantitatively different results would be obtained with different paramaterization of the AVN cell model. That said, to our knowledge the AVN cell model used in this study is the only biophysically detailed model of spontaneous AVN activity that incorporates the majority of experimental data available on rabbit AVN electrophysiology, and it has been shown to reproduce typical behaviour of AVN tissue [7], [59]. Thus, it is reasonable to propose physiological significance of I_B,Na_ as a consequence of simulations performed with this model.

## 5 Conclusions

This study demonstrates the presence of I_B,Na_ in cells from the AVN, provides additional pharmacological information on the current to that hitherto available and provides the first estimate of single channel conductance for the channel underlying cardiac I_B,Na_. Considered collectively, the data in the present study support a conclusion that I_B·Na_ is carried by a distinct low conductance NSCC, the underlying molecular basis of which remains to be established. Our simulation data provide evidence that I_B,Na_ can influence normal AVN electrophysiology (both pacemaking and conduction), whilst the current's sensitivity to reduced pH_e_ highlight this conductance as a potential target for (patho)physiological modulation. Future work should be devoted to uncovering the molecular basis of this channel, better to be able to explore its role both in myocytes from the cardiac pacemaker-conduction system and, more widely, in other regions of the heart.

## Disclosures

None.

## Appendix A Supplementary data

Supplementary material. Image 1
