# Supplementary material for: Characterization and influence of cardiac background sodium current in the atrioventricular node
Source: J Mol Cell Cardiol. 2016 Aug;97:114–24. doi: 10.1016/j.yjmcc.2016.04.014 (PMC5007024; doi:10.1016/j.yjmcc.2016.04.014)
Supplement: Supplementary file 1 — Supplementary material. [file mmc1.docx]

Characterization and influence of cardiac background sodium current in the atrioventricular node

Hongwei Cheng PhD ^1^, Jue Li PhD^2^, Andrew F. James DPhil ^1^, Shin Inada PhD^2^, Stéphanie C.M. Choisy PhD^1^, Clive H. Orchard PhD, FRSB^1^, Henggui Zhang PhD^3^, Mark R. Boyett PhD, FRSB, FRCP(hon)^2^, Jules C. Hancox PhD, FRSB, FBPhS^1*^

**Online Supplementary Information**

**Running title**: I­_B,Na_ in the atrioventricular node

*^1^ School of Physiology, Pharmacology & Neuroscience,*

*Biomedical Sciences Building,*

*University of Bristol,*

*Bristol BS8 1TD, UK*

*^2^ Institute of Cardiovascular Sciences,*

*University of Manchester,*

*Core Technology Facility,*

*46 Grafton Street,*

Manchester, M13 9NT, UK

*^3^ Department of Physics and Astronomy,*

*University of Manchester,*

*Oxford Road,*

Manchester, M13 9PL, UK

* Corresponding author. Tel.: +44-1173312292; Fax: +44-1179288923; *E-mail address:* [jules.hancox@bristol.ac.uk](mailto:jules.hancox@bristol.ac.uk) (Jules C. Hancox).

**Supplemental Methods**

*Noise analysis of current variance*

The variance of the Na^+^-sensitive current was calculated from the integral of spectral density function, as described elsewhere [1-3]. Whole-cell currents were passed through an 8-pole Bessel filter with a cut-off frequency of 2 kHz and sampled at 10 kHz. The DC component of currents was subtracted, a Hanning (cosine) window applied to the data and the power spectrum calculated using the fast Fourier transform of IgorPro 3.16B (Wavemetrics Inc, OR, USA) with appropriate re-scaling. Current traces were analyzed in blocks of 2048 data points. Thus, the lowest frequency that could be resolved was ~4.883 Hz (*i.e.* 10 kHz/2048) and the highest corresponded to the Nyquist frequency of 5 kHz. However, since the cut-off frequency of the whole cell configuration, *f*_RC_ = 1/(2πR_s_C_m_) ranged from as low as ~400 Hz, only data ranging from 4.883Hz to 400 Hz were included in the analysis. For presentation purposes, spectra were calculated from blocks of 8192 data points so that the lowest frequency resolved was ~1.221 Hz (i.e. 10 kHz/8192). The spectrum in Tris solution was assumed to represent background noise and subtracted from the spectrum in Na^+^ solution to obtain the spectrum of the Na^+^-dependent current. The Na^+^-dependent spectra were fitted by a double Lorentzian function:

**Equation S1:**  $S\left( f \right)= \frac{{S(0)}_{1}}{1+ {(\frac{f}{f_{c1}})}^{2}}+ \frac{{S(0)}_{2}}{1+({\frac{f}{f_{c2}})}^{2}}$

where S(0)_1_ and S(0)_2_ (with units of A^2^s) and *f*_c1_ and *f*_c2_ (Hz) are the low frequency asymptotes and corner frequencies of the Lorentzian components to the power spectrum.

The current variance (σ^2^) was calculated as the integral of the power spectral density. Assuming that the Na^+^-sensitive currents represented a single population of non-selective cation channels gating between a single closed state and open state with an open probability ≤ 0.1, the unitary open channel current amplitudes were calculated as:

**Equation S2:**  $i= \frac{\sigma^{2}}{I_{B,Na}}$

where *I*_B,Na_ is the Na^+^-dependent portion of the whole-cell current.

**Supplemental Results**

Additional simulations were performed in which L-type calcium current (I_Ca,L_) was set to zero, in order to induce quiescence in the AVN cell model and obtain a ‘resting’ potential for the model ‘N’ cell in the absence of spontaneous activity, but with I_B,Na_ present. Figure S1 shows the result of this simulation: in the absence of I_Ca,L_ the cell became quiescent with a resting potential of -40 mV. This is comparable to quiescence observed in real AVN cells with experimental I_Ca,L_ inhibition [4] and with reported zero current potentials under voltage-clamp for rabbit AVN cells of -40 mV or close to this potential (e.g. [5-7]).


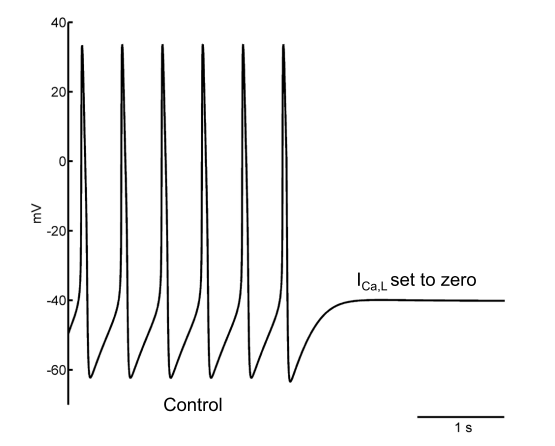


**Figure S1**  *Effect of simulated I_Ca,L_ abolition on AVN cell membrane potential*

Setting G_Ca,L_ to zero (to abolish I_Ca,L_) abolished spontaneous activity and membrane potential became quiescent at a value of -40 mV.

In contrast to quiescence induced by I_Ca,L_ abolition, removal of I_B,Na_ (Figure 7A) resulted in quiescence at a more hyperpolarized membrane potential (~-53 mV), as would be predicted from elimination of an inward (depolarizing) current.


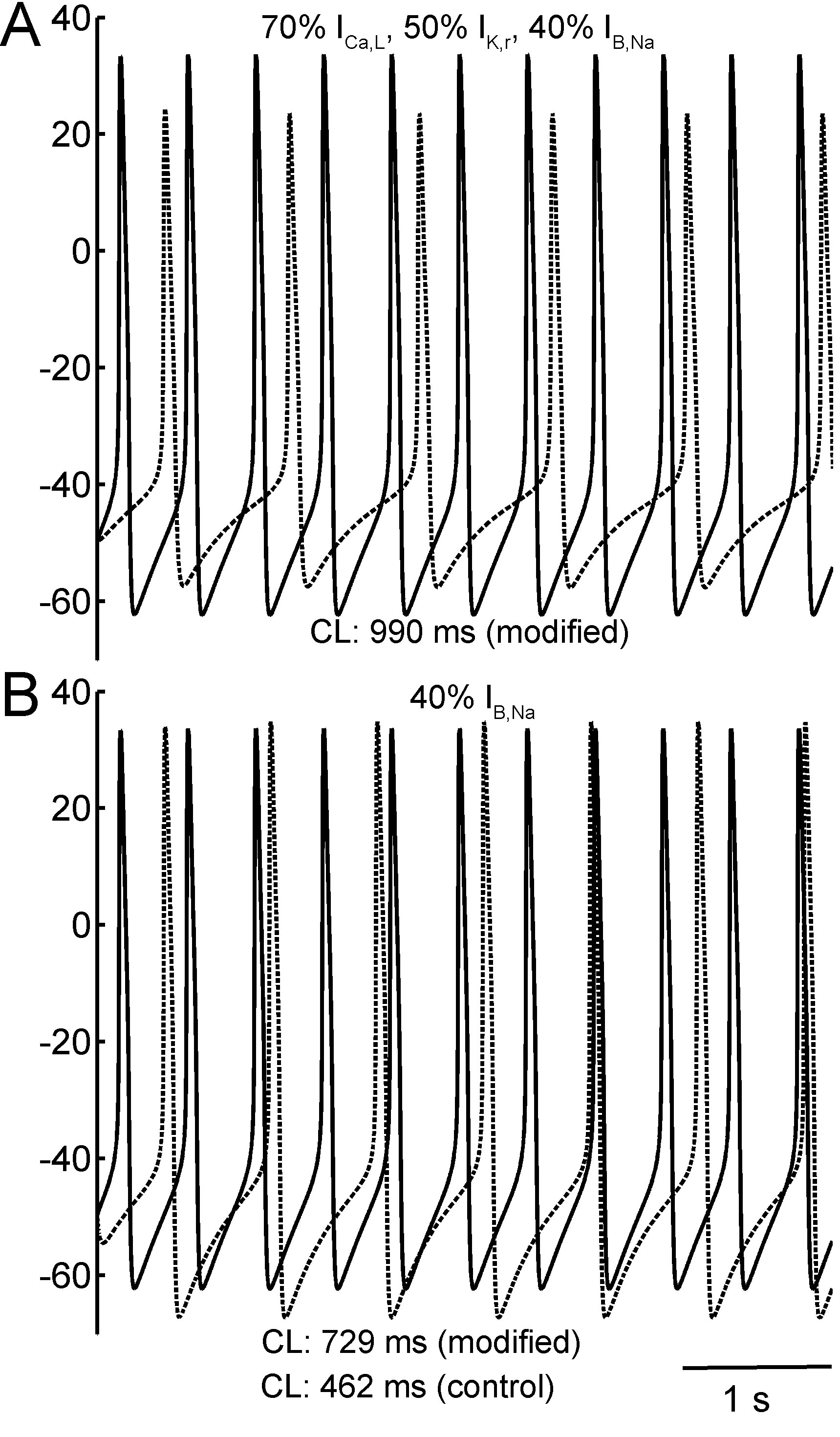


**Figure S2**  *Effect of simulated partial I_B,Na_ reduction, with and without I_Kr_ and I_Ca,L_ reduction*

**A** shows spontaneous action potentials in control conditions (solid line) and with reductions of 30% in I_Ca,L_, 50% in I_Kr_ and 60% in I_B,Na_ (dashed line). The spontaneous frequency decreased from 2.16 Hz to 1.01 Hz (CL increase from 462 ms to 990 ms), equivalent to a 53.3 % decrease in spontaneous rate.

**B** shows spontaneous action potentials in control conditions (solid line) and with a reduction of I_B,Na_ of 60% (dashed line). The spontaneous frequency decreased from 2.16 Hz to 1.37 Hz (CL increase from 462 to 729 ms), equivalent to a 36.6% decrease in spontaneous rate.

Reduction in pH_e_ from 7.4 to 6.3 has previously been reported to produce a partial slowing of AVN cell spontaneous action potential rate, together with reductions in I_Ca,L_, I_Kr_ and net background current (under non-selective recording conditions) [8]. Our data under I_B,Na_-selective recording conditions show that reduction of pH_e_ from 7.4 to 6.3 decreased inward I_B,Na_ by ~60 %. Consequently, a further set of simulations was executed to determine effects of partial I_B,Na_ reduction on spontaneous action potential rate, both with and without concomitant changes in I_Ca,L_ and I_Kr_. In AVN cell experiments, peak I_Ca,L_ was reduced by  25.3 ± 4.5% at 0 mV and 33.5 ± 7.8% at +40 mV at pH_e_ 6.3 [8]; in simulations we incorporated a 30 % I_Ca,L_ reduction. Experimentally observed reductions in I_Kr_ of 23.4 ± 8.3 % (at −30 mV) to 54.9 ± 4.7 % at (+40 mV) were seen [8]; in simulations we incorporated a 50% I_Kr_ reduction. The experimentally observed reduction in spontaneous action potential rate on reduction of pH_e_ to 6.3 was 38.5 ± 3.6 % (SEM, n=18; ± 15.3 SD) [8]. In the ‘N’ cell AVN model 60% reduction of I_B,Na_ alone reduced spontaneous action potential rate by 36.6 %, whilst combined reduction of I_B,Na,_ I_Ca,L_ and I_Kr_ reduced spontaneous action potential rate by 53.3% (within 1 SD of the experimentally observed reduction in rate), whilst also recapitulating reductions in action potential overshoot and depolarisation of maximum diastolic potential [8] (effects not seen in simulations with reduction of I_B,Na_ alone).

**References**

[1] Choisy SC, Hancox JC, Arberry LA, Reynolds AM, Shattock MJ, James AF. Evidence for a novel K^+^ channel modulated by alpha (1A) adrenoceptors in cardiac myocytes. *Molecular Pharmacology* 2004;66:735-48.

[2] Cheng HW, James AF, Foster RR, Hancox JC, Bates DO. VEGF activates receptor-operated cation channels in human microvascular endothelial cells. *Arterioscler Thromb Vasc Biol* 2006 August;26(8):1768-76.

[3] Bond RC, Choisy SC, Bryant SM, Hancox JC, James AF. Inhibition of a TREK-like K^+^ channel current by noradrenaline requires both beta1- and beta2-adrenoceptors in rat atrial myocytes. *Cardiovasc Res* 2014 October 1;104(1):206-15.

[4] Hancox JC, Levi AJ. L-type calcium current in rod- and spindle-shaped myocytes isolated from the rabbit atrioventricular node. *Am J Physiol* 1994;267:H1670-H1680.

[5] Taniguchi J, Kokubun S, Noma A, Irisawa H. Spontaneously active cells isolated from the sino-atrial and atrio-ventricular nodes of the rabbit heart. *Jpn J Physiol* 1981;31:547-58.

[6] Hancox JC, Levi AJ, Lee CO, Heap P. A method for isolating rabbit atrioventricular node myocytes which retain normal morphology and function. *Am J Physiol* 1993;265:H755-H766.

[7] Martynyuk AE, Kane KA, Cobbe SM, Rankin AC. Adenosine increases potassium conductance in isolated rabbit atrioventricular nodal myocytes. *Cardiovas Res* 1995;30(5):668-75.

[8] Cheng H, Smith GL, Orchard CH, Hancox JC. Acidosis inhibits spontaneous activity and membrane currents in myocytes isolated from the rabbit atrioventricular node. *J Mol Cell Cardiol* 2009 January;46(1):75-85.
